# Could Defatted Mealworm (*Tenebrio molitor*) and Mealworm Oil Be Used as Food Ingredients?

**DOI:** 10.3390/foods9010040

**Published:** 2020-01-02

**Authors:** Yang-Ju Son, Soo Young Choi, In-Kyeong Hwang, Chu Won Nho, Soo Hee Kim

**Affiliations:** 1 Natural Products Research Institute, Korea Institute of Science and Technology, Gangneung Institute of Natural Products, Gangneung, Gangwon-do 25451, Korea; yangjuson@kist.re.kr (Y.-J.S.); cwnho1423@kist.re.kr (C.W.N.); 2 Department of Food and Nutrition and Research Institute of Human Ecology, Seoul National University, Seoul 08826, Korea; ikhwang@snu.ac.kr; 3 Sempio, Seoul 04557, Korea; drd0603542@naver.com; 4 Department of Culinary Arts, Kyungmin University, Uijeongbu, Gyeonggi-do 11618, Korea

**Keywords:** mealworm, edible insect, defatted powder, mealworm oil, characteristics

## Abstract

Before edible insects may be used as an alternative food, it is necessary to develop basic product forms and evaluate their characteristics. We made two basic commercial products (defatted powder and oil) from mealworm, a popular edible insect. The defatted mealworm powder possessed a sufficient amount of protein, and it had a savory taste due to plentiful free amino acids. Additionally, it had abundant minor nutrients and bioactive compounds. The physicochemical properties of mealworm oil were very similar to vegetable oil, and mealworm oil was also abundant in bioactive nutrients, especially γ-tocopherol. In addition, the predicted shelf life of mealworm oil was suitable for commercial use. Moreover, mealworm had high antioxidant and anti-inflammation activities, which may arise from functional peptides and glucosamine derivatives such as chitin and chitosan. In short, the defatted mealworm powder and mealworm oil could be successfully used as novel food ingredients.

## 1. Introduction

Although people are apprehensive about consuming insects, edible insects are one of the most promising alternative protein sources. Historically, humans have consumed many types of insects across the globe. The earth has been undergoing environmental change, and one of the major causes of this has been the increase in consumption of meat from vertebrates. However, it is a challenge to replace all animal-based foods with vegetables because of the low content of proteins and limiting amino acids in plants. For this reason, vegetarians may be critically exposed to a paucity of particular vitamins and minerals. In comparison, insects possess sufficient amounts of protein, and their amino acid distribution is better than that of vegetative protein sources [1].

The mealworm (*Tenebrio molitor* L.) is an edible insect that has been popular in the animal feed industry, and mealworm-based feed products have been extensively used from pets to fish farms. Numerous studies have found that mealworm provides moderate nourishment and that there is no noxious effect of consumption [2,3,4,5]. Moreover, one of the important advantages of mealworm as a food source is that it is suitable for mass production. The mealworm is tolerant to various environmental conditions and does not require a large area for growth [6]. Additionally, the mealworm has good sensory characteristics. Their texture is crispy, and the mealworm has a savory taste that is very similar to dried shrimp.

A major problem associated with edible insects is an aversion to consumption based on their appearance. Thus, when mealworms are used in the food industry as an ingredient, processing such as pulverization and extraction could be helpful. However, the mealworm is composed of almost 30% fat, and this exceedingly high fat content is troublesome during pulverization processes. The agglomeration of powders, known as caking, is facilitated by high fat content in powders, and this state gives rise to a decrease in powder flowability [7]. For this reason, the separation of protein-rich and oil-rich components could be a good approach for developing food items from mealworms, as is the case for soybean. Hence in this study, defatted mealworm powder, and mealworm oil as a byproduct, were separated and the nutrition value and physicochemical characteristics of each were examined.

The goal of this study was to suggest proper products from mealworm that can be used in the food industry and consumed by public. Considering the psychological hesitation to eat insects, pulverization process was applied for elimination of its appearance. The defatted powder and oil were selected as basic products because the high fat content of mealworms disturbed the powdering process and transport equipment commonly used in the food industry. For defining characteristics of defatted mealworm powders, nutritional values and physicochemical characteristics were determined.

## 2. Materials and Methods

All chemicals used in this study were purchased from Sigma-Aldrich (St. Louis, MO, USA), and live mealworms were purchased from a farm in Gyeonggi-do, Korea.

### 2.1. Preparation of Mealworm Powder and Oil Samples 

Live mealworms were fasted for three days to empty their guts. Mealworms were washed with tap water three times and blanched in boiling water (1:5, *w*/*w*) for 3 min. Blanched mealworms were kept on a colander for cooling and removing excess water. After 30 min, the remaining water was removed with a paper towel. An industrial hot-air dryer (LH.FC-PO-150; Lab house, Pocheon, Korea) was used for drying mealworms (12 h, 60 °C). Then, dried mealworms were pulverized with a blender (HR-2860; Philips, Amsterdam, The Netherlands) until resulting powders could pass a 30-mesh sieve (535 μm), and this powder was used as whole-fat mealworm powder (WF-M).

For extracting oils, fivefold n-hexane was added to WF-M, and the mixture was placed on a shaker (SI600R; Lab Companion, Daejeon, Korea) for 6 h at 170 rpm. The extracted liquids were filtered with a No. 1 filter paper (Whatman, Buckinghamshire, UK). After three repetitions of extraction, n-hexane was removed with an evaporator (RE111; Bűchi, Flawil, Switzerland) at 34 °C. The remained n-hexane in oils were eliminated with nitrogen gas, and the supernatant was collected as mealworm oil after centrifugation for 10 min at 1350× *g* (Combi-514R; Hanil Science Industrial, Daejeon, Korea). A picture of mealworm oil is shown in Figure S1. The unsaponifiable lipid was made from mealworm oil by applying a saponification process, and its yield was 1.8% ± 0.0%.

After oil extraction, the powder remaining on the filter was collected. The defatted mealworm powder was put in a hood for 24 h at room temperature and further dried using a speed vacuum evaporator (Maxivac alpha; Labogene ApS, Lynge, Denmark) for 24 h at 40 °C. The pressure was adjusted to under 7 mmHg, and its rotation speed was 2000 rpm. Finally, the powder was ground once more with a blender (HR-2860; Philips, The Netherlands) until it could pass through a 42-mesh sieve (355 μm), and it was defatted the mealworm powder (DF-M) sample. The mealworm protein isolate (MPI) was produced from DF-M using the method of Sharma and Singh [8]. First, 20 g of DF-M was mixed with 200 mL of deionized water, and the pH was adjusted to pH 12 with adding 1 N NaOH. After shaking for 1 h at 220 rpm (SI600R; Lab Companion, Korea), the solution was centrifuged for 20 min at 2000× *g* (Combi-514R; Hanil Science Industrial, Korea). The supernatant was collected, and the pH of solution was adjusted for 4.5 using 1 N HCl. After centrifuging for 20 min at 2000× *g* once more, liquid part was eliminated, and residue was collected only. The MPI was prepared by lyophilizing the residue. The yield of MPI was 13.0% ± 0.3% and the crude protein content of MPI was 91.1% ± 1.1%. Pictures of mealworm powder samples are presented in Figure S2.

The 80% methanol extract was obtained from DF-M. 40 g of DF-M was put into a flask, and 80% methanol solution (1:10, *w*/*v*) was added. At room temperature, the flask was placed in a shaker (SI600R; Lab Companion, Korea) for 12 h at 200 rpm. The solvent was filtered with a No.1 filter paper (Whatman, UK) and remaining powders were extracted twice more. Collected solvents were evaporated with an evaporator (RE111; Bűchi, Switzerland), and the extraction yield was 15.5% ± 0.6%.

### 2.2. Proximate Compositions and Amino Acid Compositions

AOAC methods were used for determining moisture, crude fat, crude protein, and ash contents [9]. The carbohydrate content was calculated based on the sum of other contents. For analyzing the amino acids compositions, 0.5 g of mealworm powder was put in a flask and 20 mL of 6 N HCl was added, heated to 110 °C, and incubated for 24 h. The total volume of the solution was adjusted to 25 mL with distilled water after filtering. For derivatization of amino acids, borate buffer, o-phthaldialdehyde/2-mercaptopropionic acid, and fluorenylmethyloxycarbonyl chloride were mixed just before HPLC injection. The reversed-phase HPLC system (Ultimate 3000; Thermoscientific Dionex, MA, USA) was used for analyzing the content of amino acids, and the analysis conditions are shown in Table S1 [10].

### 2.3. Protein Solubility

The protein solubility of mealworm powder was determined with little modifications using the methods of Morr et al. [11] Distilled water was added to mealworm powder (1:10, *w*/*v*), and pH was adjusted to a range of 2–12 with 1 N NaOH and 1 N HCl. After centrifuging for 20 min at 2000× *g* (Combi-514R; Hanil Science Industrial, Korea), the supernatant was collected. The Bradford method was used for measuring the amount of protein in the supernatant, and the protein solubility at each pH was compared with the protein solubility at pH 12, the maximum point of protein solubility.

### 2.4. Gel Permeation Chromatograph Analysis

Gel permeation chromatography (GPC) was used to observe soluble particle size distributions. Mealworm powder samples were dissolved in 0.02 M NaNO_3_ (1:10, *w*/*v*) for gathering hydrophilic particles, and the supernatant was collected by centrifugation (2000× *g*, 20 min). The GPC analysis was conducted with conditions as presented in Table S2. The soluble particle sizes were obtained by comparison with a calibration plot of log (molecular weight) versus retention time, created with polystyrene standards (Figure S3) [12].

### 2.5. Fatty Acid Composition

A total of 0.25 g of mealworm oil was mixed with 6 mL of 0.5 M methanol sodium hydroxide, and heated using a water bath (80 °C, 10 min) for methylation of the oils. The mixtures were cooled on ice, and 7 mL of 14% boron trifluoride methanol solution was added, then the mixture was reheated (80 °C, 2 min). After cooling on ice, 5 mL of n-hexane was added and the mixture was again heated (80 °C, 1 min). The top layer was collected and examined for fatty acid composition. Gas chromatography (GC) was used for analysis under conditions described in Table S3 [13].

### 2.6. Physicochemical Properties of Mealworm Oil

The specific gravity of mealworm oil was determined according to ASTM D1298 using a pycnometer. Specific gravity was calculated as the ratio of gravities between that of mealworm oil and distilled water at 15 °C. The viscosity of mealworm oil at 20 °C was measured with a Brookfield DV-IP viscometer (Brookfield, Middleboro, MA, USA) using spindle No. 62 at 60 rpm. Color values were measured with a colorimeter (CM-3500d; Minolta, Tokyo, Japan). A total of 4 g of mealworm oil was put to 35-mm petri dishes, and a light source was set to D65–10°. The Hunter Lab colorimetric system was employed. The peroxide values and acid values were determined by the method of the Ministry of Food and Drug Safety of Korea [14]. The thiobarbituric acid reactive substances (TBARS) content in oil was measured using the method of Buege and Aust [15]. The induction time of oil was analyzed using rancimat 734 (Metrohm, Herisau, Switzerland). As described in the method of Gómez-Rico et al. [16], the measurement conditions were analogous to AFNOR NF EN ISO 6886. A total of 3 g of mealworm oil was put in a container, and oxidation was induced at 98 °C under 20 L/h air flow.

### 2.7. Minor Nutrients in Mealworm Oil

The contents of tocopherols in mealworm oil was measured using the method of Gliszczyńska-Świgło and Skiorska [17] with some modifications. First, 1 g of oil was dissolved in 5 mL of n-hexane and filtered with a 0.2 μm syringe filter. Reversed-phase HPLC was used with operation conditions as described in Table S4. For the determination of the content of polyphenol, 10 g of oil was dissolved in 50 mL of n-hexane, and 20 mL of 60% methanol was added. After separating layers, the methanol fractions were collected and evaporated at 40 °C. This condensed polyphenol residue was analyzed using the Folin–Ciocalteu method [18]. The content of squalene and sterols was analyzed in unsaponifiable lipid. The operating conditions for GC-MS are described in Table S5.

### 2.8. Bioactive Nutrients of Mealworm Powder and Antioxidant Capacity

γ-Aminobutyric acid (GABA) and taurine contents were analyzed using the same methods as amino acid composition analysis. Total glucosamine content was measured by the method of Belcher, Nutten, and Sambrook [19]. The content of polyphenol was determined by the Folin–Ciocalteu method [18], and the flavonoid content was measured by the method of Meda et al. [20] The polyphenol and flavonoid content was calculated with gallic acid equivalent (GAE) and catechin equivalent (CE) using standard curves, respectively. The antioxidant capacity of mealworm powder was confirmed by measuring DPPH radical scavenging activity and ABTS radical scavenging activity [21,22]. The antioxidant capacities of mealworm samples were compared with trolox standard, and were computed using trolox equivalent (TE).

### 2.9. NO Reduction in Lipopolysaccharide-Induced RAW 264.7 Cell Line

The murine macrophage cell line (RAW 264.7) was purchased from the American Type Culture Collection (ATCC, Rockville, MD, USA). The RAW 264.7 cells were seeded in a 96-well plate with a density of 5 × 10^4^ cells per well. Then, 24 h after plating, the media was removed and cells were washed with PBS. The 80% methanol extract of DF-M and the unsaponifiable lipid of mealworm oil were added to cell cultures in various concentrations at 50 μL per well. An hour later, the 50 μL of media containing lipopolysaccharide (LPS) (2 μg mL^−1^) was added to each well. To analyze nitrite production, the supernatant of each well was collected after 24 h and mixed with Griess reagent at a 1:1 ratio. After 20 min, samples were analyzed with a microplate reader (SpectraMax 190, Molecular Devices, Sunnyvale, CA, USA) at 550 nm. The concentration of nitric oxide was calculated by comparison with a sodium nitrite standard curve.

### 2.10. Statistical Analysis

All data are represented as the mean ± standard deviation (SD). Measurements were performed in triplicate. One-way analysis of variance (ANOVA) and Duncan’s multiple-range test (*p* < 0.05) were performed using the IBM SPSS statistics program, version 21.0 (IBM Inc., Armonk, NY, USA).

## 3. Results and Discussion

### 3.1. Proximate Compositions and Amino Acid Compositions of Mealworm

The mealworm is composed of high fat and high protein with low carbohydrate content (Table S6). The proximate composition of freeze dried WF-M is almost the same as that of Zhao et al. [23] The WF-M had 32.3% ± 1.0% lipid content and this content is much higher than that found in soybean or meat [24,25]. When oils were extracted with n-hexane, DF-M had 70.8% ± 5.8% protein content with 2.0% ± 0.2% lipid content. Therefore, DF-M could be used as a protein rich food source.

The amino acid composition was calculated as a ratio to the crude protein amount (Table 1). The essential amino acid and non-essential amino acid ratio (E/NE) of mealworm was lower than meats such as chicken (0.75) and beef (0.7–0.8) [26,27]. Additionally, the content of branched amino acids (BCAA) was approximately 2% less than that of beef, eggs and chicken and accounts for 20%–22% of all amino acids [26,27].

The amino acid scores were calculated with the criteria of FAO/WHO [28] (Table 2). WF-M and DF-M both indicated methionine as a limiting amino acid. The DF-M presented a higher amino acid score than WF-M more than twice, and this may be caused by the difference in the loss-ratio for different types of amino acids. In the study of Jones et al. [29], there were two essential amino acids whose amino acid values were lower than the criteria, and these amino acids were same as those identified in this study (lysine and methionine). Jones et al. [29] said that the amino acid composition of mealworm could vary significantly depending on the mealworm feed, and lysine and methionine are known to be restricted amino acids in grains such as rice and soybean. Therefore, it is deemed necessary to increase the content of methionine in mealworm feed for better amino acid scores.

### 3.2. Protein Solubility and Soluble Particle Size Distributions of Mealworm Powders

The protein solubility of DF-M and MPI over a range of pH conditions are shown in Figure S4. For DF-M, protein solubility was less than 30% at pH 7 compared to pH 12. It was expected that mealworm protein is mainly composed of hydrophobic rather than hydrophilic protein, which can cause increased turbidity in solution. Therefore, it was deemed that it would be necessary to modify or degrade mealworm protein to enhance its solubility in use. In comparison, the MPI sample had a rapid decrease in solubility only near the isoelectric point, pH 4–6. Therefore, MPI appears to be a more suitable form for use in liquid foods.

**Figure 1:**
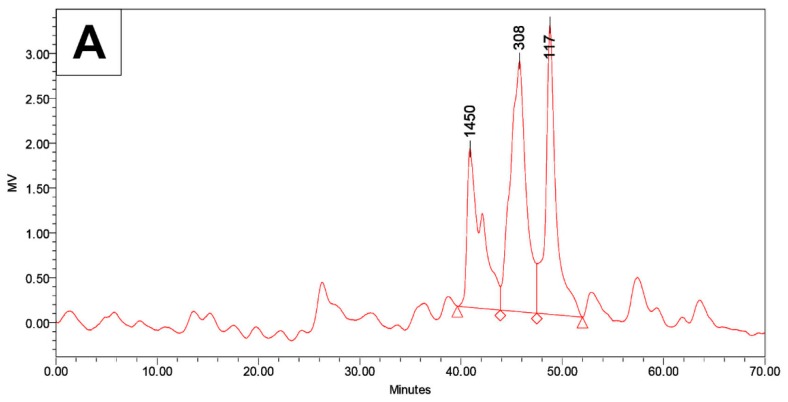

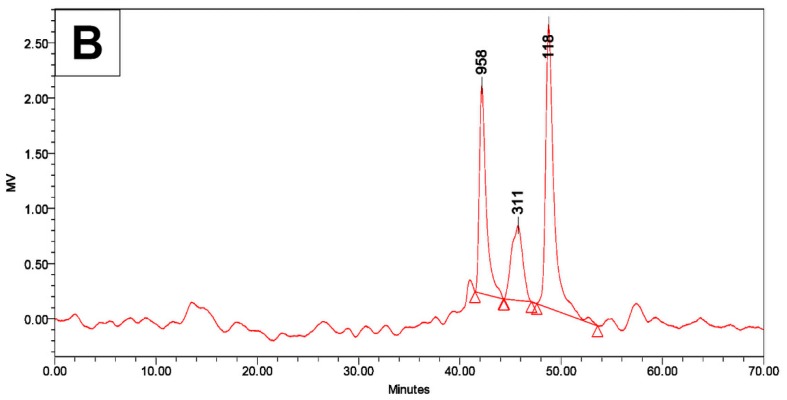
Size distribution of soluble particles of mealworm samples by gel permeation chromatography (GPC) analysis. (**A**) Defatted mealworm powder; (**B**) mealworm protein isolate.

Using gel permeation chromatography (GPC), the size distribution of soluble particles of DF-M and MPI were analyzed (Figure 1). The hydrophilic solutions of DF-M and MPI showed peaks at 117–118, 308–311, 958, and 1450 Da. As the average molecular weight of amino acids is approximately 142 Da, peaks that matched single amino acids (117–118 Da) and peptides composed of two (308–311 Da), seven (958 Da), and 10 (1450 Da) amino acids were analyzed. The most conspicuous difference in peak areas between DF-M and MPI was the peak of 308–311 Da. In DF-M, the peak area of the protein fraction with a molecular weight of 308 Da was 42.6%, but when protein was purified to MPI, the peak area of 311 Da was reduced to 18.0%. Therefore, it is assumed that a large number of particles in this range was lost during MPI refining.

### 3.3. Fatty Acid Composition of Mealworm Oil

Regarding the composition of fatty acids in mealworm oil (Table 3) oleic acid (44.5% ± 1.1%) was the most abundant fatty acid, and linoleic acid was the (19.5% ± 0.8%) second most abundant. The fatty acid composition results showed were very similar to study of Jeon et al. [13] about oil of roasted or unroasted mealworm oil. Animal lipids contain high levels of saturated fatty acids (SFA) compared to vegetable lipids. Mealworm, however, possessed a much higher amount of unsaturated fatty acid (66.0% ± 0.7%). The content of polyunsaturated fatty acid (PUFA) of mealworm (19.8% ± 0.9%) was also higher than pork (15.7%), lamb (2.3%), and beef (1.6%) [30]. In comparison, mealworm oil had a much higher value (47.0 ± 4.6) for the ratio of n-6 fatty acids and n-3 fatty acids than typical animal lipids. The high ratio of n-6: n-3 is characteristic of vegetative oils, and the ratio for corn oil (31.9) was similar to that of mealworm oil [31]. Therefore, the composition of fatty acids in mealworm oil is believed to be much closer to the characteristics of vegetable oil.

### 3.4. Physicochemical Properties and Minor Nutrients of Mealworm Oil

The physicochemical properties and amounts of minor nutrients of mealworm oil are described in Table 4. Mealworm oil appeared as a light-yellow liquid, such as commonly used edible oils, with an extraction yield of 29.5% ± 1.0%. The peroxide value of mealworm oil was higher than those of commercialized edible oils, which were in the range of less than 2 meq/1000 g oil [31]. A previous study [13] of mealworm oil showed even higher peroxide values (4.98–9.94 meq/1000 g oil) than this study, suggesting that the high peroxide value of extracted oil may be a trait of the mealworm itself.

Tocopherol (vitamin E) is one of the most important nutrients in lipids. It is a natural antioxidant that prevents free radical and hydroperoxy radical oxidation of lipids in fat-soluble foods, and it is synthesized in plants and algae [32]. In mealworm oil, the total tocopherol content was 144.3 ± 3.0 mg/1000 g oil, and γ-tocopherol was the main type of tocopherol (123.5 ± 2.7 mg/kg oil), accounting for 85.6% of the total tocopherol. In regard to animal food sources, chicken and pork contained 2.7–4.2 and 9.5–100 mg/1000 g oil total tocopherol, respectively [33,34]. In comparison, the total tocopherol amounts in olive and soybean oils are in the range of 80–1360 mg/1000 g oil [35], so the total tocopherol content of mealworm oil was lower than vegetable sources and higher than animal lipids. Polyphenols are also widely distributed in vegetable foods, and are known to have variable functionalities. The polyphenol content of mealworm oil was measured at 18.0 ± 1.3 mg/1000 g oil. This value was 10%–20% of that in olive oil and grape seed oil (62–172 mg/1000 g oil) [16]. Some vegetable oils contain abundant phytosterols, such as stigmasterol and ergosterol, and these compounds have biological activities when ingested, but neither phytosterol was detected in mealworm oil of this study. Cholesterol is mainly contained in animal foods and is known to be associated with arteriosclerosis, increasing the concentration of lipid globules in the blood. When GC-MS was used for detection of cholesterol, the chromatogram for mealworm oil had extremely small peaks, therefore, we also failed to quantify this. Therefore, mealworm oil was much richer in minor nutrients than animal lipids, and its cholesterol amount was lower than the limit of quantitation and limit of detection (LOQ ≈2.6 ppm, LOD ≈8.7 ppm).

The induction time of mealworm oil was measured by accelerating oxidation using a rancimat, and the commercial extra-virgin olive oil was used as control in this study. The induction time of mealworm oil was 36.0 ± 0.7 h, and it was lower than that of olive oil (47.6 ± 0.8 h). A higher induction time indicates that they are less oxidized. In the study of Gómez-Rico et al. [16], the induction time of commercial olive oil was in the range of 30–60 h, and this value was similar with that obtained in this study. For the viability of mealworm oil as a commercial product, we predicted the shelf life of oil based on the induction time results. In the study of Farohoosh [36], they calculated Q10 values and the oil stability index (OSI20) that offers a prediction of shelf life of oils at 20 °C based on the induction time. Under these conditions, the shelf life of olive oil in this study was forecasted to 404 d. This value was similar with shelf life of commercial extra virgin olive oil. When using this forecast model, the estimated shelf life of mealworm oil at 20 °C was 305 d, which was approximately 10 months.

### 3.5. Bioactive Nutrients, Antioxidant Capacity, and Anti-Inflammation Activity of Mealworms

In Table 5 the bioactive nutrients in DF-M are presented. In general, the amount of taurine in animal food sources is higher than that in plants. For example, beef and pork have 40–60 mg/100 g [37]. Although the amount of taurine in mealworms was about half as much as that in beef or pork, the taurine content of DF-M (17.8 ± 0.3 mg/100 g) was higher than that of plant sources. The mealworm is known to contain glucosamine derivatives in its skin, such as chitin and chitosan. Glucosamine is one of the natural amino sugars of the hexosamine family and it is a basic monomer of chitin (N-acetyl-D-glucosamine) and chitosan. Chitin and chitosan relieve degenerative arthritis by inhibiting inflammatory reactions in the joint region [38]. The total glucosamine content of DF-M was measured as 7.0% ± 0.7%.

The content of polyphenols and flavonoids in DF-M were 8.2 ± 0.2 mg GAE/100 g and 2.0 ± 0.2 mg CE/100 g, respectively, and these amounts were lower than those of vegetables such as onions, tomatoes, and cabbage, and fruits such as cherries and plums (13–100 mg GAE/100 g) [39]. However, the DPPH and ABTS radical scavenging powers of mealworm extract were not much less than vegetable foods. Thus, it was thought that there are additional substances involved in antioxidant properties besides polyphenols and flavonoids. Many studies have shown that some peptides in protein foods have excellent antioxidant properties [40]. Therefore, it is expected that some specific peptides in mealworm also affect in its antioxidant capacity.

**Figure 2:**
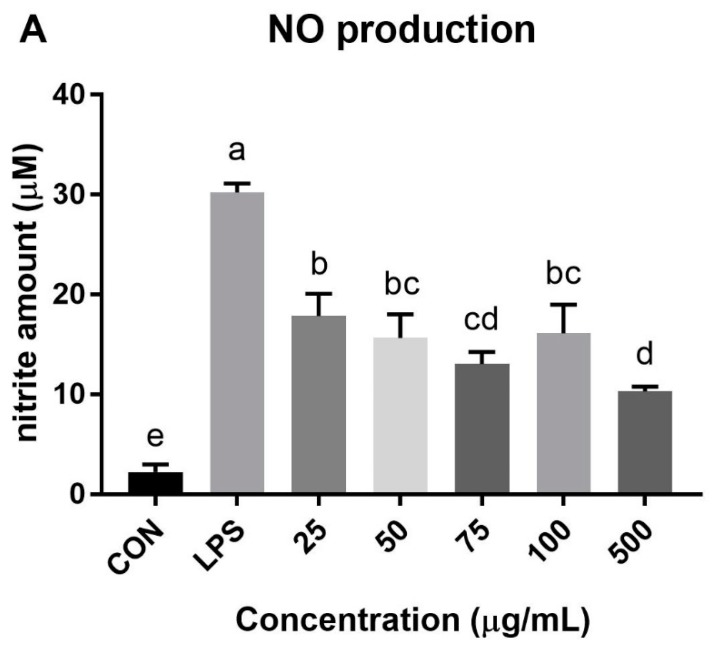

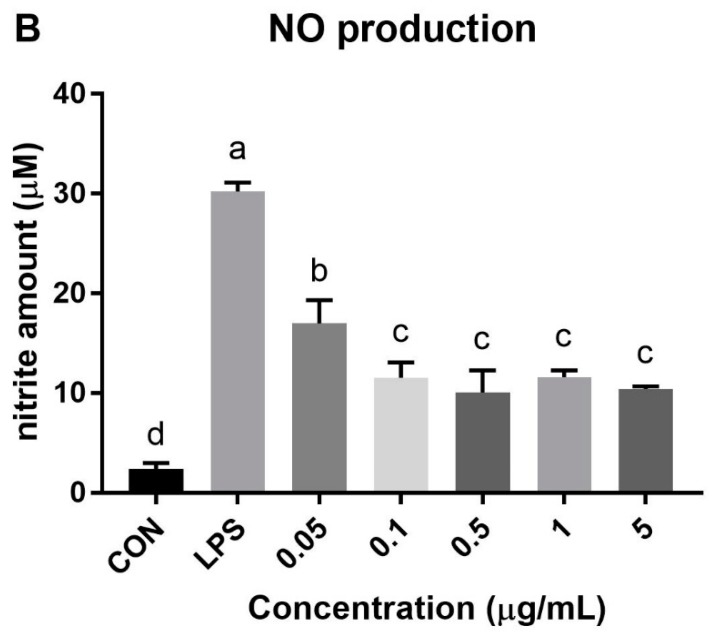
Reduce of NO production on lipopolysaccharide (LPS)-induced (1 μg/mL) RAW 264.7 cell line. (**A**) The 80% methanol extract of defatted mealworm powder; (**B**) unsaponifiable lipid of mealworm oil. Different superscripts (a–e) represent significant differences at *p* < 0.05.

In addition, as shown in Figure 2, the NO production of LPS-induced RAW 264.7 cells were lowered by treatment with 80% MeOH extract of DF-M (25–500 μg/mL) and unsaponifiable lipid (0.05–5 μg/mL). As suggested for antioxidant activities, functional peptides and glucosamine derivatives such as chitin and chitosan may strongly affect the anti-inflammation activity of mealworm extract [38,41]. Additionally, the unsaponifiable lipid of mealworm oil decreased NO production significantly, demonstrating that the lipid portion of mealworm could alleviate inflammation as well.

## 4. Conclusions

In this study, two basic types of products (defatted powder and oil) were prepared from whole fat mealworm, and both products were characterized for use as food ingredients. DF-M was light-brownish powder rich in protein (70.8% ± 5.8%). Methionine was a limiting amino acid of DF-M, and the proportion of lysine in DF-M was also lower than established criteria. Therefore, it would be necessary to increase methionine in mealworm feed in order to improve amino acid scores. Meanwhile, free amino acids were very abundant in DF-M, which could strongly affect the taste of mealworm products. The protein solubility in water was not great with DF-M, so some processing would be needed for preventing turbidity and precipitation. The refining of DF-M to MPI was able to enhance the protein solubility. For mealworm oil, oleic acid was the most abundant fatty acid, and the fatty acid composition of mealworm oil was very similar to vegetable oils. Mealworm oil contained plentiful tocopherols and other minor nutrients, but only trace levels of cholesterol. The predicted shelf life was almost 10 months. Therefore, mealworm oil has appropriate characteristics for general-purpose use. Moreover, the antioxidant capacity and anti-inflammation activity of DF-M extract were very high, likely as a result of abundant glucosamine derivatives and functional peptides. In short, defatted mealworm powder has adequate nutritional value and is rich in protein, minor nutrients, and bioactive compounds. Mealworm oil, a byproduct of DF-M, also had good nutritional value and characteristics for versatile use as a food ingredient. In addition, the DF-M and mealworm oil had antioxidant and anti-inflammation activities. Thus, mealworm could replace meat from vertebrates, but there was one limitation with respect to a specific amino acid, i.e., methionine.

## Figures and Tables

**Table 1:** Amino acids composition of whole-fat and defatted mealworm.

	WF-M ^†^	DF-M ^‡^
Protein (%)	52.2 ± 0.6	70.8 ± 5.8
Essential amino acids (g/100 g protein)		
Histidine	3.1 ± 0.0	2.9 ± 0.2
Lysine	5.1 ± 0.1	5.1 ± 0.4
Methionine	0.5 ± 0.0	1.2 ± 0.1
Phenylalanine	3.9 ± 0.0	3.9 ± 0.2
Threonine	4.8 ± 0.0	4.3 ± 0.2
Isoleucine	4.5 ± 0.0	4.5 ± 0.2
Leucine	7.5 ± 0.0	7.5 ± 0.4
Valine	6.4 ± 0.0	6.4 ± 0.4
Sub total	35.8 ± 0.0	35.6 ± 0.3
Non-essential amino acids (g/100 g protein)		
Alanine	8.1 ± 0.0	8.1 ± 0.6
Aspartic acid	8.4 ± 0.0	8.4 ± 0.4
Arginine	5.7 ± 0.0	5.7 ± 0.3
Cysteine	N.D.	N.D.
Glutamic acid	13.0 ± 0.0	13.2 ± 0.5
Glycine	5.3 ± 0.0	5.3 ± 0.4
proline	5.2 ± 0.0	5.4 ± 0.4
Serine	4.8 ± 0.0	4.8 ± 0.3
Tyrosine	7.3 ± 0.0	7.6 ± 0.5
Sub total	57.7 ± 0.0	58.5 ± 0.4
Total (E + NE) ^§^	93.5 ± 0.0	94.1 ± 0.4
E/NE	62.0	60.9
BCAA contents (%) ^¶^	19.4 ± 0.0	18.3 ± 0.4

^†^ WF-M: Blanched and hot-air dried mealworm, ^‡^ DF-M: Defatted WF-M with solvent (n-hexane), ^§^ E: essential amino acids, NE: non-essential amino acids, ^¶^ BCAA: branched amino acids.

**Table 2:** Essential amino acid amounts and amino acid scores of mealworm powders.

	FAO/WHO Ref. (1985)	WF-M ^†^	DF-M ^‡^
Histidine	20	30.7	28.9
Lysine	55	51.1	50.8
Methionine + Cysteine	35	5.1	11.5
Phenylalanine + Tyrosine	60	111.6	114.5
Threonine	40	47.9	43.3
Isoleucine	40	44.6	44.5
Leucine	70	75.4	74.5
Valine	50	63.9	64.0
Limiting amino acids		Met	Met
Amino Acids Score (AAS)		14.6	32.9

^†^ WF-M: Whole-fat mealworm powder, ^‡^ DF-M: Defatted WF-M.

**Table 3:** Fatty acid composition of mealworm oil.

Fatty Acids	Composition (g/100 g Oil)
C4:0	N.D.
C6:0	N.D.
C8:0	N.D.
C10:0	N.D.
C11:0	N.D.
C12:0	0.3 ± 0.0
C13:0	0.1 ± 0.0
C14:0	4.0 ± 0.2
C14:1_cis_	N.D.
C16:0	15.8 ± 0.1
C16:1_cis_	1.8 ± 0.1
C17:0	0.1 ± 0.0
C18:0	2.3 ± 0.2
C18:1_cis_	44.5 ± 1.1
C18:2 (n-6)	19.5 ± 0.8
C18:3 (n-3)	0.4 ± 0.1
C20:0	0.1 ± 0.0
C20:1_cis_	0.1 ± 0.0
C20:2 (n-6)	0.1 ± 0.0
Total	88.6 ± 0.6
SFA ^†^	22.6 ± 0.1
MUFA ^‡^	46.2 ± 1.6
PUFA ^§^	19.8 ± 0.9
USFA ^¶^	66.0 ± 0.7
n-6:n-3 ratio	47.0 ± 4.6
P:S ratio	0.9 ± 0.0

Data are expressed as mean ± SD, ^†^ SFA: Saturated fatty acid, ^‡^ MUFA: Monounsaturated fatty acid, ^§^ PUFA: Polyunsaturated fatty acid, ^¶^ USFA: Unsaturated fatty acid.

**Table 4:** Physicochemical properties and nutrient composition of mealworm oil.

	Mealworm Oil
Extraction Yield (%)	29.5 ± 1.0
Specific gravity (15 °C)	0.8528 ± 0.0032
Viscosity (cP)	324.2 ± 6.3
Color	
L (lightness)	38.9 ± 0.3
a (redness)	−1.9 ± 0.1
b (yellowness)	7.5 ± 0.6
Peroxide value (meq/1000 g oil)	3.5 ± 0.2
Acid value (mg KOH/g oil)	2.6 ± 0.0
TBARS (mg MDA/1000 g oil)	1.8 ± 0.1
The contents of tocopherols (mg/1000 g oil)	
α-Tocopherol	6.3 ± 0.1
β-Tocopherol	8.5 ± 0.6
γ-Tocopherol	123.5 ± 2.7
δ-Tocopherol	6.1 ± 0.3
Total tocopherol	144.3 ± 3.0
Total polyphenol (mg GAE ^†^/1000 g oil)	18.0 ± 1.3
Squalene (mg/1000 g oil)	21.1 ± 5.0
Induction time (h)	36.0 ± 0.7
Predicted shelf-life (day) ^‡^	305

Data are expressed as mean ± SD, ^†^ GAE: Gallic acid equivalent, ^‡^ Shelf-life prediction at 20 °C was calculated by OSI_20_ × (factor of safety), OSI_20_: induction time at 20 °C (calculated by Q_10_ factor: 2.05), factor of safety: 0.7.

**Table 5:** The γ-Aminobutyric acid (GABA), taurine, glucosamine, polyphenol, and flavonoid contents and radical scavenging activities of defatted mealworm.

	DF-M ^†^
GABA (mg/100 g dry basis)	3.5 ± 0.1
Taurine (mg/100 g dry basis)	17.8 ± 0.3
Glucosamine (g/100 g dry basis)	7.0 ± 0.7
Polyphenol (mg GAE ^‡^/100 g dry basis)	8.2 ± 0.2
Flavonoid (mg CE ^§^/100 g dry basis)	2.0 ± 0.2
DPPH (mg TE ^¶^/g dry basis)	21.5 ± 0.5
ABTS (mg TE ^¶^/g dry basis)	12.3 ± 0.5

Data are expressed as mean ± SD, ^†^ DF-M: Defatted mealworm powder, ^‡^ GAE: Gallic acid equivalent, ^§^ CE: Catechin equivalent, ^¶^ TE: Catechin equivalent.

## Supplementary Materials

The following are available online at https://www.mdpi.com/2304-8158/9/1/40/s1, Figure S1: Pictures of mealworm oil extracted with n-hexane, Figure S2: Pictures and color values of whole- or defatted-mealworm powders and mealworm protein isolate (MPI), Figure S3: GPC calibration plot, Figure S4: Protein solubility of mealworm powder, Table S1: HPLC operating conditions for analyzing amino acid contents, Table S2: Operating conditions for GPC analysis, Table S3: GC analysis conditions for examining fatty acids composition, Table S4: Operating conditions of HPLC for analyzing tocopherol contents, Table S5: Analysis conditions of squalene and sterols by GC-MS analysis, Table S6: Proximate compositions of mealworm powders.
