# Supplementary material for: Could Defatted Mealworm (Tenebrio molitor) and Mealworm Oil Be Used as Food Ingredients?
Source: Foods. 2020 Jan 2;9(1):40. doi: 10.3390/foods9010040 (PMC7023496; doi:10.3390/foods9010040)
Supplement: Supplementary file 1 [file foods-09-00040-s001.pdf]

Supplementary materials

Could Defatted Mealworm (*Tenebrio molitor*) and Mealworm Oil Be Used as Food Ingredients?

Yang-Ju Son [1,2], Soo Young Choi [3], In-Kyeong Hwang [2], Chu Won Nho [1] and Soo Hee Kim [4,*]

[1] Natural Products Research Institute, Korea Institute of Science and Technology, Gangneung Institute of Natural Products, Gangneung, Gangwon-do 25451, Korea
[2] Department of Food and Nutrition and Research Institute of Human Ecology, Seoul National University, Seoul 08826, Korea
[3] Sempio, Seoul 04557, Korea
[4] Department of Culinary Arts, Kyungmin University, Uijeongbu, Gyeonggi-do 11618, Korea

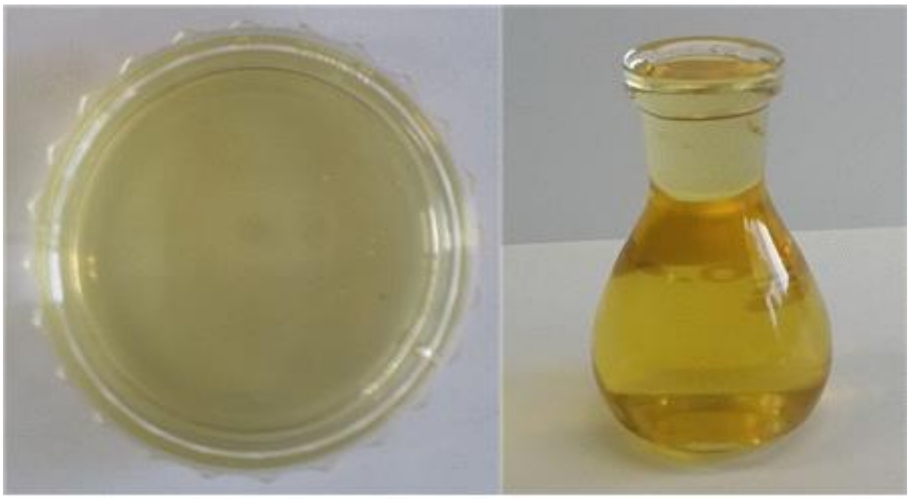

Fig. S1. Pictures of mealworm oil extracted with n-hexane

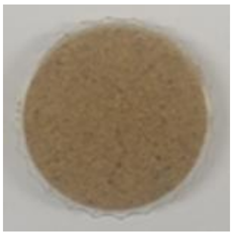

L: 36.95 ±0.16

a: 0.67 ±0.06

b: 0.54 ±0.03

WF-M

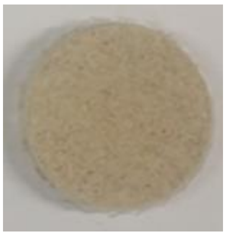

L: 71.59 ±0.62

a: 4.28 ±0.11

b: 12.28 ±0.32

DF-M

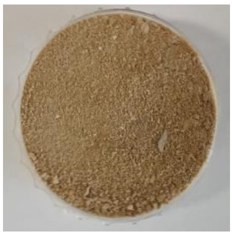

L: 63.69 ±3.29

a: 4.16 ±0.04

b: 11.53 ±0.59

MPI

Fig. S2. Pictures and color values of whole- or defatted-mealworm powders and mealworm protein isolate (MPI)

WF-M, whole-fat mealworm powder; DF-M, defatted WF-M with solvent (n-hexane); MPI, mealworm protein isolate

L, lightness; a, redness; b, yellowness

Table S1. HPLC operating conditions for the determination of amino acid content

Instrument parameter	Condition	
Model	Ultimate 3000<br>(Thermo Scientific Dionex, Waltham, MA, USA)	
Detector	1. UV detector: 338 nm<br>2. FL detector<br>Excitation: 340 nm, Emission: 450 nm (OPA)<br>Excitation: 266 nm, Emission: 305 nm (FMOC)	
Column	VDSpher 100 C 18-E<br>(4.6×150 mm, 5 μm)<br>(VDS optilab, Berlin, Germany)	
Mobile phase	A: 20 mM sodium phosphate monobasic (pH 7.8)<br>B: water/acetonitrile/methanol (10:45:45, v/v)	
Gradient condition	Time (min)	%B
	0	0
	24.0	57
	24.5	100
	26.0	100
	26.5	0
	30.0	0
Flow rate	1.5 mL/min	
Injection volume	0.5 μL	

Temperature	Column: 40℃<br>Sample: 20℃

Table S2. Operating conditions for GPC analysis

Instrument parameter	Condition
Model	Breeze 2 HPLC system (Waters, Milford, MA, USA)
Detector	RI detector
Column	Waters Ultrahydrogel linear<br>Waters Ultrahydrogel 500<br>Waters Ultrahydrogel 250<br>Waters Ultrahydrogel 120<br>(Waters, USA)
Mobile phase	0.02 N $NaNO_3$
Flow rate	0.8 mL/min
Temperature	Column: 30°C<br>Sample: 20°C

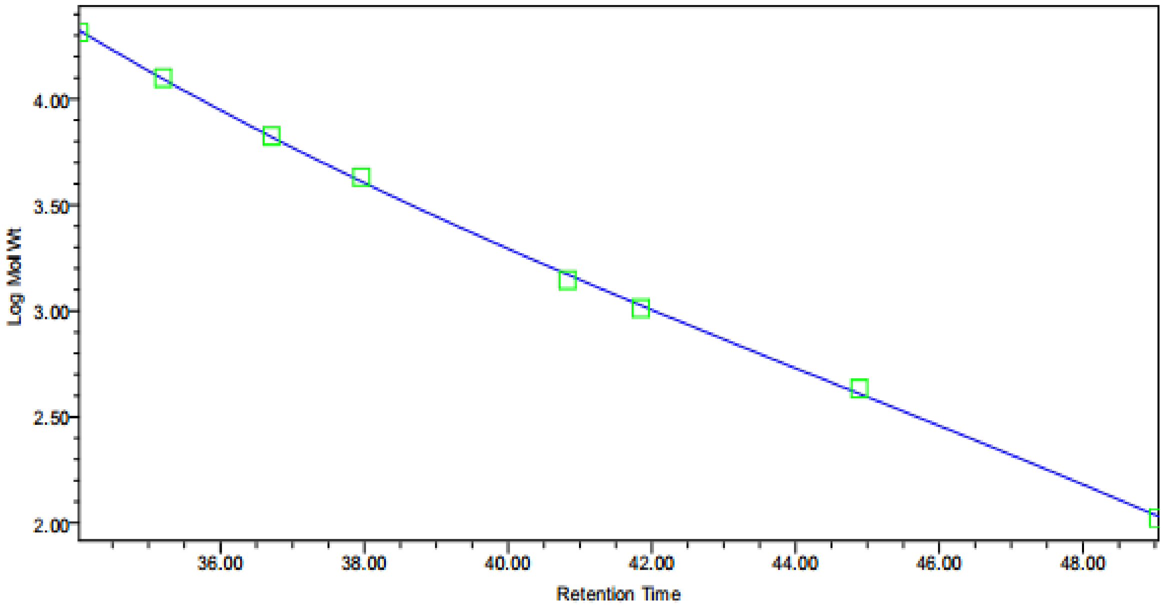

Fig. S3. GPC calibration plot

Table S3. GC analysis conditions for examining fatty acids composition

Instrument parameter	Condition
Model	Agilent 6890<br>(Agilent Technologies, SantaClara, CA, USA)
Detector	260 flame ionization detector
Column	DB-23 capillary column<br>(30 m×0.25 mm×0.25 μm)<br>(J&W Scientific, Folsom, CA, USA)
Carrier gas	Helium gas
Flow rate	1.3 mL/min
Injection volume	1 μL
Injection mode	Split mode<br>(Split ratio 50:1)

Table S4. HPLC operating conditions for the determination of tocopherol content

Instrument parameter	Condition
Model	Ultimate 3000<br>(Thermo dionex, USA)
Detector	UV detector (295 nm)
Column	C_{18} Inno Column<br>(4.6×250 mm, 5 μm)<br>(Innopia, Seongnam, Korea)
Mobile phase	100% MeOH
Gradient condition	isocratic flow
Flow rate	1.0 mL/min
Injection volume	10 μL
Temperature	Column: 20℃<br>Sample: 20℃

Table S5. Operating conditions of GC-MS for the determination of squalene and sterols

Instrument	Parameter	Condition
GC	Model	ISQ LT<br>(ThermoScientific, USA)
	Detector	260 flame ionization detector (FID) & MS
	Column	VF-5MS (30 m×0.25 mm×0.25 μm)<br>(Agilent, USA)
	Oven condition	120℃ for 5 min<br>15℃/min to 320℃<br>320℃ for 15 min
	Carrier gas	Helium gas
	Flow rate	1.5 mL/min
	Injection volume	1 μL
	Injector temperature	200℃
	Injection mode	Splitless mode
MS	Mass range	35-550 Da
	Transfer line temperature	250℃
	Ion source temperature	250℃
	Analyzer	Quadrupole Single MS-PMT<br>(Photomultiplier detector)
	Aquisition mode	MS scan and SIM
	Ion source	Electron Ionization (EI)

Table S6. Proximate compositions of mealworm powders

(unit: %)

	Moisture	Crude protein	Crude lipid	Ash	Carbohydrate
Blanched	61.5±0.5[a2)]	20.5±1.3[c]	13.5±0.8[b]	1.2±0.0[c]	3.4±0.2[c]
WF-M[1)]	0.5±0.1[c]	52.2±0.6[b]	32.3±1.0[a]	3.6±0.0[b]	11.5±0.4[b]
DF-M	4.0±0.9[b]	70.8±5.8[a]	2.0±0.2[c]	5.1±0.1[a]	18.2±3.8[a]

Data are expressed as mean±SD

[1)]WF-M: Blanched and hot-air dried whole-fat mealworm, DF-M: Defatted WF-M with solvent (n-hexane)

[2)]Different superscripts within columns (a-c) represent statistically significantly differences at $p<0.05$ by Duncan's multiple range test

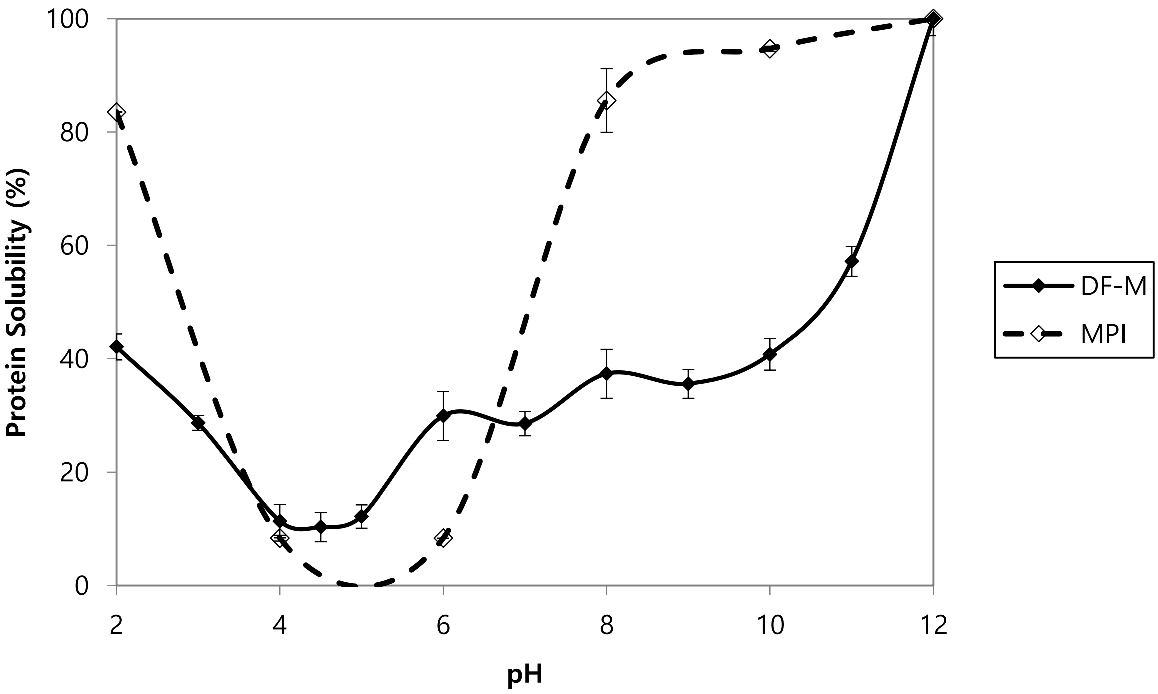

Fig. S4. Protein solubility of mealworm powder

DF-M, Defatted mealworm powder; MPI, Mealworm protein isolate
